# Supplementary material for: Long-distance spin-transport across the Morin phase transition up to room temperature in ultra-low damping single crystals of the antiferromagnet α-Fe2O3
Source: Nat Commun. 2020 Dec 10;11:6332. doi: 10.1038/s41467-020-20155-7 (PMC7729397; doi:10.1038/s41467-020-20155-7)
Supplement: Supplementary file 1 — Supplementary Information [file 41467_2020_20155_MOESM1_ESM.pdf]

Supplementary Information: Long-distance spin-transport across the Morin phase transition up to room temperature in ultra-low damping single crystals of the antiferromagnet α-Fe_2O_3

R. Lebrun,[1,2] A. Ross,[2,3] O. Gomonay,[2] V. Baltz,[4] U. Ebels,[4] A-L. Barra,[5] A. Qaiumzadeh,[6] A. Brataas,[6] J. Sinova,[2,7] and M. Kläui[2,3,6]

[1] *Unité Mixte de Physique CNRS, Thales, Université Paris-Saclay, 91767, Palaiseau, France*

[2] *Institut für Physik, Johannes Gutenberg Universität Mainz, D-55099 Mainz, Germany*

[3] *Graduate School of Excellence Materials Science in Mainz (MAINZ), Staudingerweg 9, D-55128, Mainz, Germany*

[4] *Université Grenoble Alpes, CNRS, CEA, Grenoble INP, SPINTEC, F-38000 Grenoble, France*

[5] *Laboratoire National des Champs Magnétiques Intenses, CNRS-UGA-UPS-INSA-EMFL, F-38042 Grenoble, France*

[6] *Center for Quantum Spintronics, Department of Physics, Norwegian University of Science and Technology, Trondheim, Norway*

[7] *Institute of Physics ASCR, v.v.i., Cukrovarnicka 10, 162 53 Praha 6 Czech Republic*

I. SUPPLEMENTARY DISCUSSION

In this section we describe the phenomenological model of magnon transport as a function of the magnetic anisotropies of hematite controlled by the temperature T and the external magnetic field $\mathbf{H}$.

A. Model

The magnetic state is characterized by the Néel vector $\mathbf{n}(T, \mathbf{H})$ whose dynamics is calculated from the dynamical equation

$$\mathbf{n} \times \left[\ddot{\mathbf{n}} - 2\gamma \dot{\mathbf{n}} \times \mathbf{H} + \gamma \alpha_G H_{\text{ex}} \dot{\mathbf{n}} - c^2 \Delta \mathbf{n} + \gamma^2 H_{\text{ex}} M_s \frac{\partial w_{\text{AF}}}{\partial \mathbf{n}} \right] = \gamma^2 H_{\text{ex}} \mathbf{n} \times \mathbf{H}_{\text{curr}} \times \mathbf{n}. \quad (1)$$

Here c is the limiting velocity of the magnons, γ is the gyromagnetic ratio, α_G isthe Gilbert damping, H_{ex} is the exchange field, which keeps the magnetic sublattice moments antiparallel, $\mathbf{H}_{\text{curr}} = \hbar \varepsilon \theta_H \mathbf{j} \times \hat{z}/(2ed_{\text{AF}} M_s)$ is directed along the spin polarization $\mathbf{p}$ ($|\mathbf{p}| = 1$) of the current in the Pt electrode, $\mathbf{j}$ is the current density, $\hbar$ is the Planck constant, d_{AF} is the penetration depth of spin current into hematite, $0 < \varepsilon \leq 1$ is the spin-polarization efficiency, θ_H is the spin Hall angle, e is the electron charge, and $M_s = |\mathbf{n}|$. The expression for the magnetic energy density $w_{\text{AF}}(\mathbf{n}; T, \mathbf{H})$ in the presence of the constant external magnetic field $\mathbf{H}$ reads (for the details of derivation, see Ref.[1]):

$$w_{\text{AF}}(T, \mathbf{H}) = M_s \left[-\frac{1}{2} H_{2\|}(T) n_Z^2 + \frac{(\mathbf{H} \cdot \mathbf{n})^2}{2H_{\text{ex}}} - \frac{H_{\text{DMI}}}{H_{\text{ex}}} \mathbf{n} \cdot \mathbf{H} \times \hat{Z} \right.$$
$$\left. - \frac{1}{6} H_{6\text{an}} \left(n_X^2 - n_Y^2 \right) \left[4 \left(n_X^2 - n_Y^2 \right)^2 - 3 \right] \right], \quad (2)$$

where $H_{\text{DMI}} > 0$ is the homogeneous DMI field responsible for a small spin canting (and finite magnetization) in the easy-plane phase, $H_{6\text{an}} > 0$ is the in-plane anisotropy associated with the rhombohedral symmetry of hematite. The out-of-plane anisotropy $H_{2\|}(T)$ depends on the temperature[1,2]. It is positive in the easy-axis phase and changes sign at the Morin temperature ($H_{2\|}(T_M) = 0$). The coordinate system is related with the crystallographic axes with Z aligned along easy axis below the Morin temperature and X is parallel to one of the in-plane easy axes above T_M. Note, that the sample-related coordinates used in the experimental setup (Fig.1a and 2a of the main text) are rotated by the angle $\psi = 33°$ around the Y axis, so that $x = Z \cos\psi + X \sin\psi$, $z = -X \cos\psi + Z \sin\psi$.

Magnons are small fluctuations $\delta\mathbf{n}$ of the Néel vector on top of an equilibrium state $\mathbf{n}^{(0)}$: $\mathbf{n} = \mathbf{n}^{(0)} + \delta\mathbf{n}$, $\delta\mathbf{n} \perp \mathbf{n}^{(0)}$. The equilibrium state is assumed to be homogeneous. The equilibrium orientation of the Néel vector $\mathbf{n}^{(0)}(T, \mathbf{H})$ is calculated by minimization of the energy (2) with the constant $|\mathbf{n}| = M_s$. Magnon spectra are calculated from the linearised Eq. (1) assuming that $\delta\mathbf{n}(t, \mathbf{k}) \propto \exp\left(-i\omega t + i\mathbf{k}\cdot\mathbf{r}\right)$.

This approach is appropriate for magons with $\mathbf{k}$ vectors far from the Brillouin zone edge which give the main contribution to the observed spin transport.

The spin polarization of an eigenmode is a vector parallel to the dynamical magnetization[3]

$$\mathbf{m}_{\rm dyn} = \frac{\delta\mathbf{n}\times\delta\dot{\mathbf{n}}}{\gamma H_{\rm ex} M_s^2}. \tag{3}$$

From the orthogonality condition $\delta\mathbf{n} \perp \mathbf{n}^{(0)}$ it follows that $\mathbf{m}_{\rm dyn} \| \mathbf{n}^{(0)}$. We consider only modes with stationary magnetization, as only these modes contribute to spin transport signal.

As follows from Eq. (3), magnetization (and spin) depends on the polarization of the magnon mode. We introduce the polarization $0 \le s \le 1$ as the ellipticity of the mode, as will be specified below (see Eq. (5)). The maximal polarization corresponds to circularly polarized modes with an ellipticity $s = 1$. The minimal polarization corresponds to linearly polarized modes with an ellipticity $s = 0$.

B. Magnon modes in absence of current

In this section, we calculate the magnon spectra in absence of the external spin current and dissipation. Figure S1 shows the phase diagram (T, H) of hematite calculated for $\mathbf{H}\|\hat{x}$ (see Fig. 1a of the main text). We introduce the line $H_{\rm crit}(T)$ which separates the regions with $\mathbf{n}^{(0)} \perp \mathbf{H}$ (above the line, region II) and with $\mathbf{n}^{(0)}$ having nonzero projection on the direction of the magnetic field $\mathbf{H}$ (below the line, region I). This corresponds to the second-order phase transition in which the magnetic field induces smooth reorientation of the Néel vector from the equilibrium orientation $\mathbf{n}_1$ at $H = 0$ to $\mathbf{n}_2 \perp \mathbf{H}$ at $H \ge H_{\rm crit}(T)$ (see pictorials in Fig. S1).

In region I the linearized equations (1) for magnon modes take the form:

$$\begin{aligned} &\delta\ddot{n}_1 - 2\omega_H\delta\dot{n}_2 - c^2\Delta\delta n_1 + \omega_1^2\delta n_1 = 0, \\ &\delta\ddot{n}_2 + 2\omega_H\delta\dot{n}_1 - c^2\Delta\delta n_2 + \omega_2^2\delta n_2 = 0, \end{aligned} \tag{4}$$

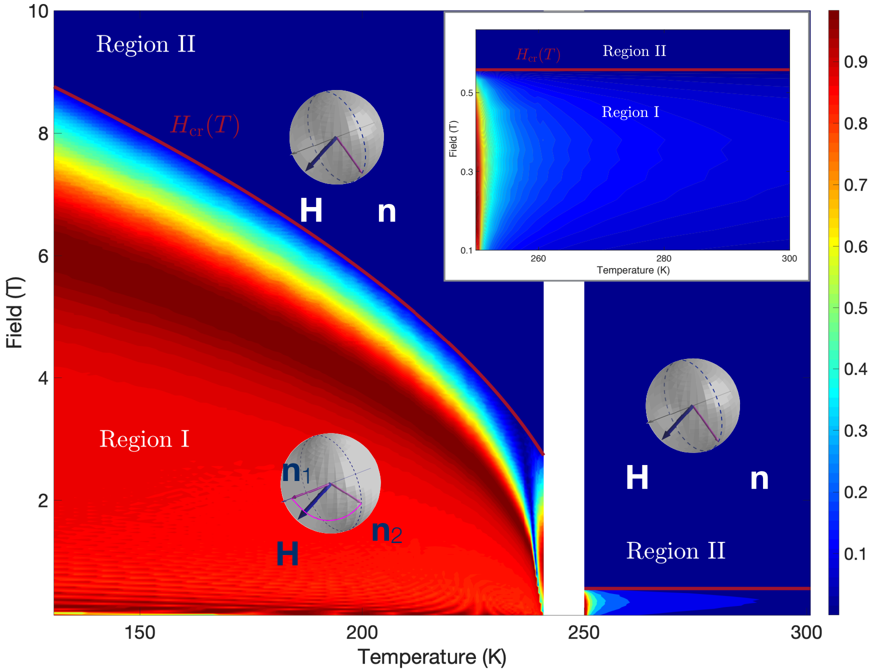

FIG. S1. The phase diagram of hematite and polarization s of magnon mode (indicated by the color code). The critical line $H_{\rm crit}(T)$ separates regions with the elliptically polarized ($s > 0$, region I) and linearly polarized ($s = 0$, region II) magnon modes. The orientation of the Néel vector in region I depends on the orientation of the magnetic field. In region II $\mathbf{n}^{(0)} \perp \mathbf{H}$. The close vicinity of the Morin temperature ($H_{2\|}(T_M) = 0$) is excluded from calculations (white stripe). The inset shows the phase diagram above the Morin temperature on the large scale. In calculations we used the data, and, in particular, the temperature dependence $H_{2\|}(T)$ obtained in Ref.[1] .

where $\delta n_{1,2}$ and $\omega_{1,2}^2$ are the eigen-vectors and eigen-values of the matrix $\gamma^2 H_{\rm ex} M_s \left(\partial^2 w_{\rm AF}/\partial n_j \partial n_k\right)|_{\mathbf{n}_0}$, $\omega_H = \gamma \mathbf{H}\cdot\mathbf{n}^{(0)}$. As follows from Eqs. (4) the eigen-modes in presence of the magnetic field are spin-polarized with field-dependent ellipticity

$$s_\pm = \frac{4\omega_H \omega_\pm \left(\omega_\pm^2 - \omega_1^2\right)}{\left(\omega_\pm^2 - \omega_1^2\right)^2 + 4\omega_H^2 \omega_\pm^2}, \tag{5}$$

where

$$\omega_\pm^2 = \frac{1}{2}\left(\omega_1^2 + \omega_2^2\right) + 2\omega_H^2 \pm \sqrt{\frac{1}{4}\left(\omega_1^2 - \omega_2^2\right)^2 + 2\omega_H^2\left(\omega_1^2 + \omega_2^2\right) + 4\omega_H^4} \tag{6}$$

are eigen-frequencies of the spin-polarized modes with $\mathbf{k} = 0$. Frequencies of the modes with nonzero $\mathbf{k}$ ore obtained by substitution $\omega_{1,2}^2 \to \omega_{1,2}^2 + c^2\mathbf{k}^2$.

In the present geometry, all eigenmodes in region I are spin-polarized due to the influence of the magnetic field, both below (in the easy-axis phase) and above (in the easy-plane)

the Morin transition temperature. The polarization degree (ellipticity) is proportional to the component of the Néel vector along the field direction and vanishes in region II. This also means that the magnon gas can be spin-polarized even in the absence of the external spin-current. An exception is the case of $H = 0$ below the Morin temperature, where the eigenfrequencies are degenerate and the eigenmodes are circularly polarized with $s = 1$.

Ellipticity of magnon modes (shown with the colour code in Fig. S1) achieves the maximal value close but slightly below the phase transition line $H_{\rm crit}(T)$. However, ellipticity substantially diminishes in the region below the Morin point where the AF is formally still uniaxial, but the value of uniaxial anisotropy is small and comparable with the in-plane anisotropy. In this case a very small magnetic field induces an orthorhombic anisotropy, suppresses elliptical polarization of magnons and makes spin transport less efficient thus reducing the amplitude of the signal.This shows up as the peak in the simulated temperature dependence of the voltage signal which appears below the Morin temperature (grey curve in Fig.1c of the main text).

In region II $\omega_H = 0$ and the magnon eigenmodes in the absence of the spin-current are linearly polarized and carry no spin.

C. Magnons in the presence of a spin current

To study the effect of the external spin current on magnons we linearise Eq. (1) assuming that $\mathbf{H}_{\rm curr}\|\mathbf{n}^{(0)}$. In region I, the corresponding equations become:

$$\begin{aligned}
&\delta\ddot{n}_1 - 2\omega_H\delta\dot{n}_2 + \gamma\alpha_G H_{\rm ex}\delta\dot{n}_1 - c^2\Delta\delta n_1 + \omega_1^2\delta n_1 = \gamma^2 H_{\rm ex}H_{\rm curr}\delta n_2,\\
&\delta\ddot{n}_2 + 2\omega_H\delta\dot{n}_1 + \gamma\alpha_G H_{\rm ex}\delta\dot{n}_2 - c^2\Delta\delta n_2 + \omega_2^2\delta n_2 = -\gamma^2 H_{\rm ex}H_{\rm curr}\delta n_1.
\end{aligned} \tag{7}$$

From Eqs. (7) it follows that the spin current modifies the effective damping coefficient of the spin-polarized modes:

$$\alpha_G^{\rm eff} = \alpha_G \pm \frac{\gamma s_\pm}{\omega_\pm}(\mathbf{H}_{\rm curr}\cdot\mathbf{n}^{(0)}) \tag{8}$$

Opposite signs correspond to the modes polarized along/opposite to the spin current.

According to the fluctuation-dissipation theorem, the modification of the effective damping given by Eq.(8) can be viewed as a modification of the effective temperature for each magnon mode[4]

$$T_\pm = \frac{T}{1 \mp \gamma s_\pm(\mathbf{H}_{\rm curr}\cdot\mathbf{n}^{(0)})/(\alpha_G\omega_\pm)}. \tag{9}$$

As a result, the spin current creates a nonequilibrium spin accumulation $\boldsymbol{\mu}$ (thermodynamically conjugate variable to magnon spin) which assuming small fluctuations can be calculated, using a standard approach (see, e.g.,[5]):

$$\boldsymbol{\mu} = \frac{\gamma}{\alpha_G}\mathbf{n}^{(0)}(\mathbf{H}_{\rm curr}\cdot\mathbf{n}^{(0)})\left[s_+ f\left(\frac{\hbar\omega_+}{T}\right) + s_- f\left(\frac{\hbar\omega_-}{T}\right)\right], \qquad (10)$$

where $f(\varepsilon) = [\exp(\varepsilon/(k_BT)) - 1]^{-1}$ is the Bose-Einstein equilibrium distribution function for each of the modes, $\hbar$ is the Planck constant, k_B is the Boltzmann constant.

In region II, where $\omega_H = 0$, and $\omega_1 \neq \omega_2$, we take into account the spacial dependence of the spin current. The orthogonal components $\delta n_1(\mathbf{k}_1)$ and $\delta n_2(\mathbf{k}_2)$ are coupled through the Fourier component $H_{\rm curr}(\mathbf{k}_1 - \mathbf{k}_2)$ of the current, as follows from the equations:

$$\delta\ddot{n}_1(\mathbf{k}_1) + \gamma\alpha_G H_{\rm ex}\delta\dot{n}_1(\mathbf{k}_1) + (\omega_1^2 + c^2k_1^2)\delta n_1(\mathbf{k}_1) = \gamma^2 H_{\rm ex}\int H_{\rm curr}(\mathbf{k}_1 - \mathbf{k}_2)\delta n_2(\mathbf{k}_2)d\mathbf{k}_2, \qquad (11)$$

$$\delta\ddot{n}_2(\mathbf{k}_2) + \gamma\alpha_G H_{\rm ex}\delta\dot{n}_2(\mathbf{k}_2) + (\omega_2^2 + c^2k_2^2)\delta n_2(\mathbf{k}_2) = -\gamma^2 H_{\rm ex}\int H_{\rm curr}(\mathbf{k}_2 - \mathbf{k}_1)\delta n_1(\mathbf{k}_1)d\mathbf{k}_1.$$

From Eq. (11) it follows that the spin current not only creates a nonequilibrium distribution of magnons, but also modifies the structure of the magnon modes. In particular, instead of linearly polarized modes , the modes in the presence of a spin current can be circularly polarized along $\mathbf{n}^{(0)}$ with $s = 1$. Such modes can be presented as a linear superposition of equilibrium modes $\delta n_1(\mathbf{k}_1)$ and $\delta n_2(\mathbf{k}_2)$ with the same frequency, satisfying the relation $\omega_1^2 + c^2k_1^2 = \omega_2^2 + c^2k_2^2$. The effective damping and temperature of these modes are renormalised similar to Eqs. (8), (9), and the renormalisation is proportional to the Fourier component $H_{\rm curr}(\mathbf{k}_1 - \mathbf{k}_2)$.

To estimate the Fourier spectrum $H_{\rm curr}(\mathbf{k}_1 - \mathbf{k}_2)$ we assume that within the yz plane the current-induced spin accumulation is homogeneously distributed within the rectangular region $-w/2 < y < w/2$, $-\ell < z < 0$, where w ($\propto 300$ nm) is the width of Pt electrode and ℓ ($\propto 1$ nm) is spin penetration depth. This gives a wide distribution of $\Delta k_z \propto 3$ nm^{-1} in the z direction and a relatively narrow distribution $\Delta k_y \propto 10$ μm^{-1} in the direction of the spin transport measurement.

The circularly polarized modes with different $\mathbf{k}$ vectors decay in space due to the dephasing effect. The characteristic length of the dephasing L can be estimated from the dispersion. We consider the mode with $\mathbf{k}_1 = (0, k_y, 0)$ and $\mathbf{k}_2 = (0, k_y, k_z)$, so that the magnon spin dephases in the z direction while the magnons propagate in the y direction.

Taking into account that the group velocity $\mathbf{v}_{\rm gr} = c^2\mathbf{k}/\omega$, so that $v_z k_y = v_y k_z$, we obtain for the propagation length in the y direction:

$$L = \frac{2\pi k_y}{k_z^2} = \frac{2\pi c^2 k_y}{\omega_1^2 - \omega_2^2}. \tag{12}$$

D. Spin-transport data for different inter-stripe distances

In this section we present the temperature dependence for the spin-transport data obtained for an inter-stripe distance of 7μm. The decrease of the decay length with temperature leads to disappearance of a significant spin-transport signal above 240 K for this distance. Furthermore, we show the field dependence of the spin-transport signal at room temperature for inter-stripe distances of 800 nm and 1 μm, which look similar as the data for 500 nm shown in the main text.

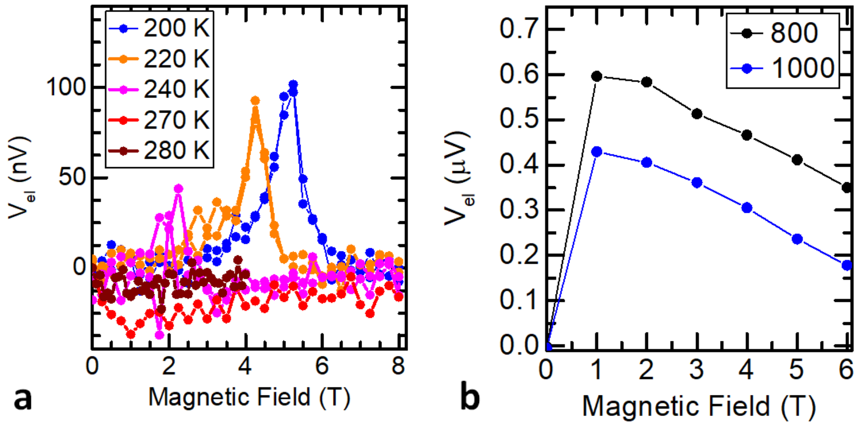

FIG. S2. (a) Spin-transport through the Morin transition ($T_{\rm M}$). Temperature dependence of the nonlocal spin-signals for a magnetic field parallel to the platinum stripes (33° from the easy-axis) for inter-stripe distances of 7 μm. (b) Field dependency of the spin-transport signal at room temperature for interstripe distances of 800 nm and 1 μm.

[1] Lebrun, R. *et al.* Anisotropies and magnetic phase transitions in insulating antiferromagnets determined by a Spin-Hall magnetoresistance probe. *Comm. Phys.* **2**, 50 (2019).

[2] Velikov, L. & Rudashevski, E. Antiferromagnetic Resonance in Hematite in the Weakly Ferromagnetic State. *Sov. J. of Experimental and Theoretical Physics* **29**, 836 (1969).

[3] Gomonay, O., Yamamoto, K. & Sinova, J. Spin caloric effects in antiferromagnets assisted by an external spin current. *J. Appl. Phys.* **51**, 264004 (2018).

[4] Gomonay, O. & Loktev, V. Spin torque antiferromagnetic nanooscillator in the presence of magnetic noise. *Conden. Matt. Phys.* **15**, 43703 (2012).

[5] Kovalev, A. A. & Tserkovnyak, Y. Thermomagnonic spin transfer and Peltier effects in insulating magnets. *EPL* **97**, 67002 (2012).
